# Usability of a mobile application for health professionals in home care services: a user-centered approach

**DOI:** 10.1038/s41598-023-29640-7

**Published:** 2023-02-14

**Authors:** Griselda Manzano-Monfort, Guillermo Paluzie, Mercedes Díaz-Gegúndez, Carolina Chabrera

**Affiliations:** 1 Home Care Services, Corporació de Salut del Maresme i la Selva, Calella, Barcelona, Spain; 2 Health Information Management Department, Corporació de Salut del Maresme i la Selva, Calella, Barcelona, Spain; 3 Tecnocampus, Universitat Pompeu Fabra, Barcelona, Spain; 4 Research Group on Chronic Care and Innovation in Health (GRACIS), Barcelona, Spain

**Keywords:** Health care, Medical research

## Abstract

The use of mobile devices by healthcare professionals has led to rapid growth in the development of mobile healthcare applications designed to improve healthcare services. This study was conducted to assess the acceptability and usability of a mobile application for health professionals in relation to their work in hospitalization at home. A mixed methods approach was used. Acceptance, included the satisfaction of the professionals, attitudes toward using the application, and intention or willingness to continue using the application. Usability tests were performed in laboratory analyzing five controlled clinical tasks, and the interaction of the participants with the mobile application was based on the six basic facial expressions published by the American Psychological Association. Perceived satisfaction was assessed using the computer system usability questionnaire. Thirty-two participants completed the task scenarios and questionnaire. More than 90 per cent of participants were able to complete the tasks with only some difficult with vital signs. Satisfaction had a score of 6.18/7 (SD: 0.76), and recommendation of the mobile application had a score of 6.21/7 (SD: 0.81). This study showed a significant usability and acceptability of this mobile application, in terms of effectiveness, efficiency, and satisfaction.

## Introduction

Mobile health (mHealth), or the use of mobile devices in medicine and health, is a sub-category of eHealth^1^. Health interventions are designed to improve healthcare services, and they may be divided into different areas, including medical records and communication^2,3^. We find three main applications. Mobile electronic health records (EHRs) used by healthcare professionals. Personal health record applications that patients can use to examine and control their own health data. And applications that allow direct patient control over records of specific diseases. The use of EHRs is expected to lead to improved efficiency, better communication, improved accessibility, and enhanced quality of care^4–7^. These services and applications that utilize mobile functionality are actively being developed in hospitals, organizations, and other groups^8–12^.

Several research studies have been performed on mHealth applications, and the results have indicated that well-designed mHealth applications can empower patients, improve medication adherence, and reduce the cost of health care^13,14^. To assess and improve upon the usability of mHealth applications, a wide range of usability evaluation methods (UEMs) are available to detect problems in user–system interaction. Employing multiple methods enables a more comprehensive assessment of the usability of eHealth interventions than using a single evaluation method^15^. The UEMs allow the identification of those facets of the interaction that need improvement^16–18^. To determine the usability of any new technology, appropriate and rigorously developed measures must be employed^19–22^. Although the use of mHealth has increased rapidly in recent decades, there is limited scientific evidence supporting its effectiveness^23–29^, possibly because of a lack of reliable information regarding proven benefits^30,31^.

### Study context

The mobile health-app that is the focus of this study, AppADIm (Mobile Integrated Healthcare at Home), was originally developed with the aim that the health professionals (doctors and nurses) of home hospitalization units could have secure access to patients’ relevant medical information as clinical notes, records of vital signs and medical orders. Record follow-up data at home, and automatically upload data to the hospital EHR, thereby saving the professionals’ time and avoiding transcription errors. Our previous study results on the developed AppADIm observed that 86% of the professionals used it on a regular basis and considered it an improvement for their daily work. The total theoretically saved hours in medical information transcription were 256 per year, which would correspond to 36.5 days (7-h shifts). The conclusion was that using an application to consult and update a patient’s health record at home avoids transcription errors and saves professionals’ time^32^. AppADIm has been evolucionating during the last years and a second version is currently in use. Although the mobile application represents an important advance and an improvement in the care provided by professionals, it is currently not being used homogeneously by all health professionals and, consequently, paper documentation is still being used during home visits. This means that patient data and records continue to be duplicated, which is a waste of time and does not sufficiently improve clinical practice or patient safety.

The aim of this study was to assess the acceptability and usability of the AppADIm for health professionals working with patients’ electronic records at home and to suggest further improvements to the application.

## Methods

### Study design

In this study, different methodologies and techniques were used to evaluate the acceptability and usability of the mobile application, which is already described in the literature^31–33^. Usability is defined as “the extent to which a product can be used by specific users to achieve specific goals with effectiveness, efficiency and satisfaction in a specific context of use”^34^. Acceptance, for the purpose of the study, included the satisfaction of the professionals, attitudes toward using the application, and intention or willingness to continue using the application^35^.

This study was conducted in three phases: Phase (A) Researchers developed an ad hoc questionnaire to explore the use of new technologies. Phase (B) Tests of the usability of the mobile application were performed by the participants while the interaction of the participants with the mobile application was analyzed using the "Think-aloud" approach and facial gesturing, with a categorical approach, based on the six basic facial expressions published by the American Psychological Association (happiness, surprise, fear, disgust, anger, and sadness)^36^. Phase (C) Using the computer system usability questionnaire (CSUQ)^37^, user-perceived satisfaction in aspects related to the ease of use, ease of learning, simplicity, effectiveness, information, and user interface of the mobile application were assessed.

### Recruitment

Participants were selected through an open call The study was carried out with professionals who were unfamiliar with AppADIm. Candidates from different areas of healthcare and with different years of care experience were included. All of them were identified with an ID to ensure confidentiality. Medical professionals, nurses, and health professionals from different areas of care, such as hospitals, health centers, geriatric residences, home care, and others, were included. All health professionals who had worked with a mobile healthcare data management application comparable to AppADIm were excluded from the study to make the sample more homogeneous in relation to the use of this technology. Thirty-two participants were included in the study and one candidate was excluded^38^, which is like the number employed in previous studies assessing the acceptance and usability of health apps^39,40^.

### Data Collection in the three phases

#### Phase A: socio-demographic data and the use of new technologies

Before evaluating the mobile application, the 32 participants completed an online questionnaire, via Google forms, regarding socio-demographic data and entailed general questions as years of experience, training, field of work, personal use of internet and the use of new technologies, developed by the authors based on the recommendations described in the bibliography and validated by a panel of experts.

#### Phase B: mobile application usability testing

The usability tests of this study were performed at the Center of Simulation and Innovation in Health (CSIS), which is a center dependent on the School of Health Sciences of Tecnocampus, located in the Tecnocampus Science Park. The participants individually performed the usability tests of the mobile application in a room equipped with a filming system. During the tests, the participants completed the tasks that two researchers were presenting from an adjoining room. The tasks evaluated in the usability tests are shown in Table 1. The criteria were tested according to the usability measures proposed in the ISO standard 9241–11^41,42^. The evaluation followed a specific order to ensure that every user had an individual perspective of each of the tasks to be performed. During the procedure, each participant’s performance was recorded with cameras at different angles, and the researchers observed the reactions and movements from the adjoining room through a double mirror. Simultaneously, mobile phone screens were recorded using an external camera, which provided images or screen recordings (Multimedia appendix 1). Participants were asked to voice any feelings, doubts, or limitations they experienced during the exercise (think-aloud) to supplement the information received. The researchers registered all aspects directly related to the effectiveness and efficiency of the participants and, subsequently, analyzed the interaction of the participants with the mobile application through facial gesturing, with a categorical approach, using the six basic facial expressions.

**Table 1:** Tasks evaluated in the usability tests.

Task number	Description of task
Task 1	Access the application, identify yourself, search the list of patients, and select a specific patient
Task 2	View the patient's personal data, verify the patient’s identity, and search for their personal address
Task 3	Review the patient’s diet and medication allergies
Task 4	Consult and register a clinical note
Task 5	Consult and record vital signs
Task 6	Consult and verify the prescribed medical orders of the admitted patient

#### Phase C: CSUQ

Finally, all participants completed the CSUQ^37^. This is the Spanish adaptation of the post-study system usability questionnaire^43^. The CSUQ consists of 16 items rated on a 7-point scale (strongly disagree^1^ to strongly agree^7^), and a general satisfaction scale and three subscales: system utility (items 1–6), information quality (items 7–12), and interface quality (items 13–15). Higher scores indicate better usability.

### Data analysis

Data analysis was based on audio and video recordings collected by cameras. The voice reactions of the participants in the audio recordings were transcribed verbatim. Incident notes, characterized by comments, silences, or repeated actions, and error messages, were collected through the recordings. The obtained content was analyzed by two members of the research team. Transcripts and critical incidents were also reviewed to identify the most common usability concerns. In any case of discrepancy in content analysis, a third-party reviewer was consulted. The results of the CSUQ questionnaire were analyzed using the statistical program Jamovi. A descriptive, inferential, and univariate study was conducted. In the univariate analysis, the quantitative variables were expressed as centralization and dispersion parameters (mean, standard deviation, etc.), and as qualitative variables, via frequencies and percentages.

### Ethical approval

The study was conducted in accordance with the Declaration of Helsinki. The study was approved by the Ethical Committee of the School of Health Sciences of Tecnocampus (CODE: 33/18).

### Consent to participate

Participants signed informed consent forms. To ensure confidentiality, only the principal investigator had access to the identity data. The results obtained will be maintained for five years.

## Results

Thirty-two participants completed the task scenarios and questionnaire. The main characteristics of the participants are summarized in Table 2. The majority were female and nurses with a high percentage of postgraduate training and the most (68,5%) had at least 10 years of experience. Almost half of participants (46,9%) had the hospital ward as working area and 75% of participants used at least one mobile health application.

**Table 2:** Characteristics of participants (N = 32).

Variable		Participants
Age in years, mean (SD)		38.8 (10.4)
Gender, n (%)	Male	8/32 (25.0)
	Female	24/32 (75.0)
Studies, n (%)	Nursing	29/32 (90.6)
	Medicine	3/32 (9.4)
Postgraduate training, n (%)	Postgraduate studies/specialization masters	13/32 (40.6)
	Official masters	12/32 (37.5)
	Doctoral degree	7/32 (21.9)
Years of care experience, n (%)	0–4	5/32 (15.6)
	5–10	5/32 (15.6)
	11–15	3/32 (9.4)
	16–20	7/32 (21.9)
	More than 21	12/32 (37.5)
Area of care, n (%)	Specialized care (hospital care)	26/32 (81.3)
	Primary health care	2/32 (6.2)
	Social/healthcare	4/32 (12.5)
Working area, n (%)	Management	1/32 (3.1)
	Primary care for adults, Primary care for children, Primary home care	2/32 (6.2)
	External consultations	2/32 (6.2)
	Convalescence/long stay/hospital palliative care	4/32 (12.5)
	Conventional hospitalization (internal medicine, surgery, traumatology)	15/32 (46.9)
	Hospitalization at home	1/32 (3.1)
	Intra-hospital emergencies, Extra-hospital emergencies	7/32 (21.9)
Number of mobile health applications used, n (%)	None	8/32 (25.0)
	1–2	16/32(50.0)
	3–4	6/32(18.8)
	5–6	1/32 (3.1)
	6–8	0 (0)
	More than 9	1/32 (3.1)

Table 3 shows the effectiveness of participants that were able to complete the task, the efficiency—i.e., whether end-users can locate the resources using the quickest and most direct route through the application—which is measured by the number of “additional” clicks required for the actions, and the time that participants need to complete the tasks, compared to an expert user.

**Table 3:** Effectiveness of participants, efficiency of the application and efficiency comparing participant and expert user.

Task number	Description of task	Effectiveness. Participants that were able to complete the task, n (%)	Efficiency. Average number of additional clicks that participants needed to complete the task	Efficiency. Average participant time/average expert user time (seconds)
Task 1	Access the application, identify yourself, search the list of patients, and select a specific patient	30/32 (93.8)	1.1	38.29/16.70 = 2.2
Task 2	View the patient's personal data, verify the patient’s identity, and search for their personal address	31/32 (96.9)	0.3	17.59/6.92 = 2.5
Task 3	Review the patient's diet and medication allergies	31/32 (96.9)	0.5	13.45/6.93 = 1.9
Task 4	Consult and register a clinical note	32/32 (100)	0.1	145.57/80.88 = 1.7
Task 5	Consult and record vital signs	27/32 (84.4)	4.2	99.99/52.21 = 1.9
Task 6	Consult and verify the prescribed medical orders of the admitted patient	31/32 (96.9)	0.0	17.95/17.17 = 1.0

More than 90 per cent of participants were able to complete the task with only some difficult with vital signs (task 5). The number of additional clicks needed was one or less except for the task 5 (vital signs) where participants did more than four. The participants used two times clicks than an expert user.

Various comments were made during the thinking-aloud process. Of the 14 comments recorded, 78.6% (11/14) were related to the task of consulting and recording vital signs (task 5).

**Figure 1:**
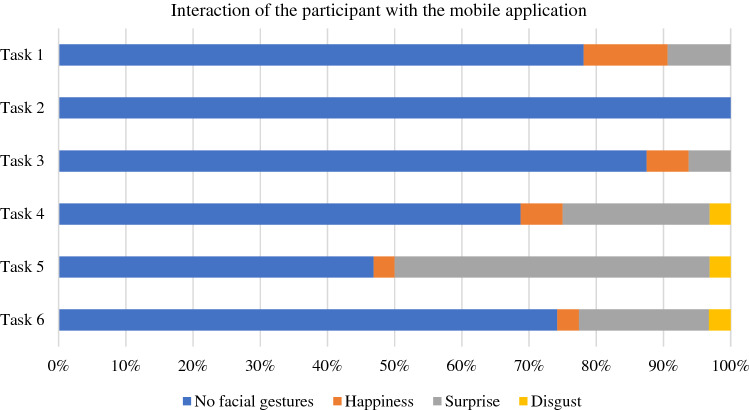
Interaction of the participant with the mobile application.

Figure 1 shows the interaction of the participants with the application. Most of the surprised reactions were noted during task 5 (consulting and recording vital signs, 50% (16/32)), followed by task 4 (consult and register a clinical note, 31% (10/32)), and task 6 (consult and verify the prescribed medical orders of the patient, 25% (8/32)).

The results from the CSUQ show that the participants were, overall, satisfied with the usability of the application (see Table 4 for details), as can be seen in the general questions section of the questionnaire. *Overall, I was satisfied with the mobile application* had a score of 6.18/7 (SD: 0.76), and *I would recommend the use of the mobile application to other professionals* had a score of 6.21/7 (SD: 0.81). Regarding the system quality, information quality, and interface quality, the best-rated category was the quality of the interface, with an average score of 6.04/7 (SD: 0.22), and the lowest rated was the quality of the information, with an average score of 5.35/7 (SD: 0.90).

**Table 4:** Computer system usability questionnaire (N = 32).

CSUQ^a^ constructs	Score^b^, mean (SD)
System quality	5.93 (0.25)
Information quality	5.35 (0.90)
Interface quality	6.04 (0.22)

^a^*CSUQ* Computer system usability questionnaire.

^b^Score range from 1 = strongly disagree to 7 = strongly agree.

## Discussion

The use of UEMs during the development and testing process of health applications is commonly recommended in the literature^44,45^. Consequently, this study aimed to critically appraise the acceptability and usability of the aforenoted mobile application for health professionals using different available UEMs to detect problems in user–system interactions and to suggest improvements to the application.

Usability tests have shown that the mobile application is efficient (which is measured by the number of “additional” clicks required for the actions and the time that participants need to complete the tasks, compared to an expert user) and effective (which is measured by the percentage of tasks completed). This is because most of the participants did not experience any difficulties performing most of the tasks with the application; moreover, only a few errors were encountered, and the time required to complete a task was comparable to that of an expert participant. This is considered an accomplishment because none of the participants had previously used the application. The most difficult task for the participants was to consult and record vital signs. In addition, most comments during the thinking-aloud process, as well as the tasks wherein the participants interacted significantly with the mobile application through facial gesturing, were also related to consulting and recording vital signs.

Overall, in this study, end-users found the mobile application to be highly usable, as indicated by the survey data (CSUQ), with no major bugs and no issues with the flow of activities. In addition, most participants expressed satisfaction with the mobile application and would recommend the use of the mobile application to other professionals.

These results suggest that the quality of the information provided with the application should be improved, and that the main task to be improved in terms of accessibility and ease of use is the consultation and registration of clinical notes of treatment. Analyzing the results obtained in a broader sense, we observe that the acceptance and satisfaction of the study participants who do not use the mobile application daily is high, like those obtained for professionals who do use it as a professional tool^32^. This suggests that, in addition to improving specific aspects of the application, a broader analysis should be performed regarding the reasons for the current limited use of the application among all professionals and the preferred use of paper for queries and to record clinical data in a complementary manner. Moreover, in the field of the Hospitalization at Home we need to take in account the aspect of the communication network. Sometimes the use of or non-use of a mobile application are related to weak network services in the area.

Some researchers have posited that one of the reasons that might explain the low usage rates, resistance, rejection of health information technology, and the request for alternative methods is that in the adoption of mobile applications and technologies, functional features and advanced techniques are prioritized, whereas the needs and characteristics of the end-users are neglected^46,47^. Other studies show that the most influential factor in the use of mobile applications is performance expectancy^48^, which is understood as the degree to which the user expects that the system will help them attain gains in job performance. Other researchers have stated that the determining factors are the perceived importance of information security, process orientation, documentation intensity, and eHealth-related knowledge^49^. Therefore, healthcare organizations should, in addition to designing and developing mobile applications that guarantee evidence-based health informatics^50^ and the utilization of UEMs, also consider performance expectancy as a determining factor in the adoption of new mobile devices; additionally, they should thoroughly analyze the end-users’ needs to identify useful functions for their workflows^51^.

### Limitations

The limitations of the present study include the sample size, although other studies have used similar or lower samples^52^, and the more presence of the nurse related to the doctor participants. Moreover, the study design did not allow for “learnability” to be measured because of the small sample size and the high efficiency and effectiveness of task scenario completion.

## Conclusions

There is clear scientific evidence for the ability of mobile handheld technology to positively impact rapid response, transcription error prevention, information accessibility, and data management in healthcare settings, as well as the beneficial impact of this technology on aspects of healthcare delivery^53^. This study has shown that the usability of this mobile application, in terms of effectiveness, efficiency, and satisfaction, is significant; however, it is not the only criterion that favors its use in daily practice. Therefore, as other scholars have also noted, further studies are needed to explore the significant antecedents of this mobile application, i.e., system and information quality and the limitations of mobile devices^46^. Future directions may include improving data integration into the health care system, an interoperable application platform allowing access to electronic health record data, cloud-based personal health records across health care networks, and increasing mobile application prescription by health care providers^2^.

## Supplementary Information

Supplementary Information.

## Data Availability

All data generated or analysed during this study are included in this published article [and its supplementary information files].

## Abbreviations

CSIS: Center of Simulation and Innovation in Health
CSUQ: Computer system usability questionnaire
EHRs: Electronic health records
mHealth: Mobile health
UEMs: Usability evaluation methods
