# Supplementary figures and images for: Usability of a mobile application for health professionals in home care services: a user-centered approach
Source: Sci Rep. 2023 Feb 14;13:2607. doi: 10.1038/s41598-023-29640-7 (PMC9929220; doi:10.1038/s41598-023-29640-7)

**Multimedia Appendix 1**

**
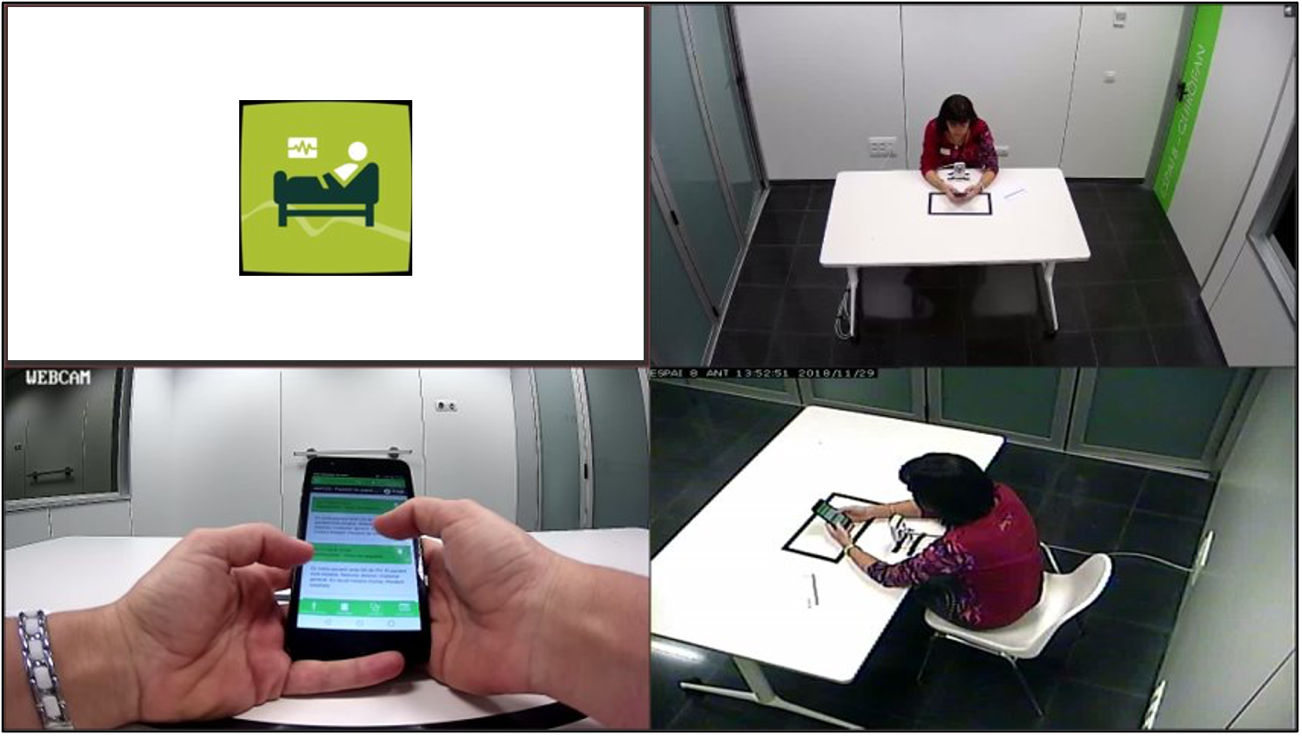
**

Multimedia appendix 1. Participant performing usability tasks.

Supplement: Supplementary file 1 — Supplementary Information. [file 41598_2023_29640_MOESM1_ESM.docx]
